# THE WORST PART OF THE DAY: DAILY REFLECTIONS OF DEMENTIA FAMILY CAREGIVERS

**DOI:** 10.1093/geroni/igac059.2552

**Published:** 2022-12-20

**Authors:** Chunhong Xiao, Vicki Winstead, Carolyn Pickering

**Affiliations:** The University of Alabama at Birmingham, Birmingham, Alabama, United States; The University of Alabama at Birmingham, School of Nursing, Birmingham, Alabama, United States; The University of Alabama at Birmingham, Birmingham, Alabama, United States

## Abstract

Family caregivers struggle with multi-dimensional demands in caring for persons living with dementia (PLWD). The challenges of caregiving combined with the requirements of daily life can impact the care of PLWD and the health and well-being of the caregivers. The purpose of this study was to describe caregivers’ perceptions of the worst part of their day in the context of daily caregiving challenges. Family caregivers completed online surveys reporting on various parts of their day. The survey included an optional open-ended question: “what was the worst part of your day?” Caregivers (N=165) completed diaries for 21 days resulting in 1,773 surveys that included a response to the optional open-ended question. A subset of data was analyzed using content analysis to identify initial codes and themes; further content analysis of the complete dataset was used to confirm and refine the identified codes and themes. Final analysis revealed 6 themes describing caregivers’ perceptions of the worst part of their day. These themes included days in which they had to: 1) cope with changes in their relationship with the PLWD, 2) manage their own health-related issues such as illness and lack of sleep, 3) struggle when there was a lack of help or support, 4) deal with daily life demands in the home along with the demands of caregiving, 5) cope with negative emotions such as sadness, grief, or anger over the disease process, 6) cope with physical exhaustion. The findings reflect daily stressors associated with caregiving for PLWD.

